# Changes of campus tobacco control environment and the impact on tobacco control behaviors among secondary school personnel in Shanghai, China

**DOI:** 10.18332/tid/191763

**Published:** 2024-09-20

**Authors:** Nuo Chen, Luojia Dai, Juanjuan Wang, Lulu Zhang, Jingfen Zhu

**Affiliations:** 1 School of Public Health, Shanghai Jiao Tong University, Shanghai, People's Republic of China

**Keywords:** secondary schools, school personnel, tobacco control environment, tobacco control behaviors, secondhand smoke

## Abstract

**INTRODUCTION:**

Youth smoking is a serious public health problem. Nevertheless, a rigorous tobacco-free environment within schools, combined with exemplary tobacco control behavior among school personnel can effectively contribute to reducing adolescent smoking. This study compared the tobacco control environment in Shanghai secondary schools in 2017 and 2021, and explored how the tobacco control environment influenced the tobacco control behaviors of school personnel.

**METHODS:**

Two cross-sectional studies were conducted from October to December 2017 and October to December 2021, using stratified cluster random sampling method, and 2403 and 1761 valid questionnaires were collected, respectively. The chi-squared test was used to test the differences between categorical variables. Binary logistic regression was conducted using survey data from 2021 to explore the influencing factors of staff’s tobacco control behaviors.

**RESULTS:**

Compared with 2017, the percentages of staff members who were current smokers, had smoked on campus in the past year and were exposed to secondhand smoke (SHS) on campus in the past 7 days in 2021 decreased by 2.95%, 2.30% and 8.91%, respectively. However, the proportion of personnel who knew the school had organized tobacco control education decreased. Furthermore, school personnel who had received tobacco control education and agreed the school should strictly prohibit students from smoking (AOR=1.64; 95% CI: 1.25–2.15) were more likely to inform about the harm of tobacco to students. Those who had participated in tobacco control education activities or tobacco control trainings (AOR=1.87; 95% CI: 1.30–2.69) and believed that the school did not strictly prohibit either students (AOR=0.30; 95% CI: 0.22–0.41) or personnel (AOR=0.46; 95% CI: 0.36–0.59) from smoking were more inclined to stop students from smoking.

**CONCLUSIONS:**

Compared with 2017, the rates of smoking and secondhand smoke exposure among school personnel decreased in 2021, but some schools still lacked comprehensive education on tobacco control behaviors for the staff. Enhancing the health literacy and strengthening tobacco control education among staff were effective strategies to encouraging their active adoption of tobacco control behaviors.

## INTRODUCTION

Smoking is a significant global public health concern, with profound implications for human well-being. In 2019, smoking was responsible for 7.69 million deaths worldwide, ranking as the primary risk factor for male mortality^1^. Research consistently has highlighted that most smokers take up this habit during adolescence, a crucial phase of growth and development^2,3^. This not only poses serious risks to both physical and mental health^4,5^, but also increases the likelihood of becoming a regular smoker in adulthood and reduces the chances of successfully quitting^6^. Therefore, controlling adolescent smoking is pivotal to the overall tobacco control efforts. Regrettably, despite ongoing efforts, adolescent smoking remains a pressing global issue. According to a report by the World Health Organization (WHO) in 2021, there were 37 million current smokers among adolescents aged 13–15 years worldwide^7^. Additionally, there were urgent issues related to tobacco use among this age group, including the rising usage of novel tobacco products (e.g. electronic cigarettes) and alarmingly high rates of exposure to secondhand smoke (SHS) in public places^8-10^.

The school environment is crucial in curbing adolescent smoking. Studies consistently show that schools that rigorously enforced tobacco control policies and strived to create smoke-free environments exhibited lower rates of adolescent smoking^11,12^. Conversely, exposure to SHS in school settings would have increased their risk of smoking initiation^13,14^. Consequently, many countries had proactively implemented a series of tobacco control measures to establish smoke-free schools. In Europe, for example, all secondary schools have adopted smoke-free school policies (SFSPs)^15^. Similarly, the United States, Canada and South Korea have also explicitly banned smoking in schools^16-19^. However, it was concerning that the establishment of smoke-free schools has not been universally achieved. Studies indicate that a substantial number of adolescents had been exposed to SHS at schools in the past 7 days, with rates varying by country (e.g. US, 16.8%^20^; and South Korea, 11.8% of boys and 11.6% of girls^21)^.

Since the promulgation and implementation of the Outline of the Health China 2030 Plan (2016), China has issued several significant tobacco control policies, such as the Notice on Further Strengthening Tobacco Control for Youth (2019) and the Notice on Further Strengthening the Construction of Smoke-Free Schools (2020). The revised Law on the Protection of Minors in 2021 explicitly banned all forms of smoking in schools, kindergartens and other public places frequented by minors. A smoke-free school environment encompassed the implementation of tobacco control system, the tobacco control education for school personnel, and the promotion of tobacco control behaviors among school personnel, etc. Nevertheless, there has been a paucity of research that evaluates the actual impact of these tobacco control laws and regulations on the tobacco control environment within schools.

School personnel, as leaders and mentors of students, play a pivotal role in student tobacco control efforts. The tobacco control behaviors of staff, such as discouraging students from smoking and advising them to quit smoking, could directly contribute to reducing the smoking rates among students^22^. Additionally, the tobacco control curriculum they deliver could indirectly prevent students from smoking by influencing students’ perceptions and attitudes toward tobacco^14,23^. Some studies indicated that the tobacco control environment in schools could significantly influence both the smoking behaviors and tobacco control behaviors of school personnel. In schools with robust tobacco control systems, staff members were less likely to smoke^24^, and they were more inclined to provide students with tobacco control information and promote smoking cessation behaviors^22,25,26^.

While previous studies have primarily focused on the impact of school tobacco control environment on students’ smoking behavior, there has been limited exploration into the influence of the school tobacco control environment on school personnel’s tobacco control behaviors. Furthermore, existing research has predominantly centered on tobacco control policies or training programs^22,25^, and there is a dearth of comprehensive analyses of multiple factors affecting school personnel’s tobacco control behaviors within the school tobacco control environment.

Therefore, this study aimed to conduct a comprehensive evaluation of the tobacco control environment within schools, including smoking rates and SHS exposure in schools, the situation of prohibiting students and school personnel from smoking, tobacco control education for school personnel, and the tobacco control behaviors exhibited by school personnel. By comparing the tobacco control environment in Shanghai’s secondary schools in 2017 and 2021, we sought to assess the effectiveness of Chinese tobacco control policies implemented after 2017. Furthermore, the study explored the impact of smoking policies and tobacco control education on school personnel’s tobacco control behaviors.

## METHODS

### Data sources

A stratified cluster randomized sampling method was used to conduct two cross-sectional studies from October to December both in 2017 and 2021. We selected 21 secondary schools from four districts in Shanghai based on the proportion of students in each school. A total of 2403 and 1761 valid questionnaires were collected in 2017 and 2021, respectively, with corresponding effective response rates of 96.47% and 97.08%.

The questionnaires were administered to all staff members of the selected schools. Both surveys were conducted anonymously on the questionnaire survey platform with the informed consent of all respondents.

### Variables

*Demographic characteristics*

The sociodemographic characteristics included gender (male/female), age group (<40 and ≥40 years), school type (traditional school/vocational school), education level (college or lower/Bachelor’s or higher; ‘college or lower’ represented incomplete undergraduate studies, completed vocational studies, or lower levels, while ‘Bachelor’s or higher’ represented obtaining a Bachelor’s, Master’s, or doctoral degree), position (teacher/non-teaching staff), whether responsible for work related to health education (yes/no), and whether smoked in the past 30 days (yes/no). Traditional schools included middle schools and high schools, while vocational schools referred to schools that taught skills needed for particular jobs. Those who smoked in the past 30 days were identified as current smokers^27^.

*Smoking and exposure to SHS among school personnel in schools*

Respondents were asked two questions: 1) ‘Have you smoked (traditional cigarettes) on campus in the past year?’ and 2) ‘Has anyone smoked (traditional cigarettes) around you on campus in the past 7 days?’. Respondents could choose ‘Yes’ or ‘No’. Those who reported that nobody smoked (traditional cigarettes) around them on campus in the past 7 days were categorized as ‘not been exposed to secondhand smoke (SHS) on campus’, while others were categorized as ‘had been exposed to SHS on campus’.

*The situation of prohibiting students and school personnel from smoking*

To assess the situation of prohibiting students and school personnel from smoking, respondents were asked two questions: 1) ‘Do you agree that the school should strictly prohibit students from smoking?’ and 2) ‘Do you agree that the school should strictly prohibit personnel from smoking?’. Respondents could choose ‘Yes’ or ‘No’.

*Tobacco control education for school personnel*

To evaluate the tobacco control education to personnel, respondents were asked the following questions: In the past year: 1) ‘Have you received any tobacco control materials from the school?’, 2) ‘How many times have you participated in tobacco control trainings organized by your school?’, 3) ‘Did your school organized any tobacco control education activities?’; and 4) ‘How many times have you participated in tobacco control education activities organized by your school?’. Response options for questions 1) and 3) were ‘Yes’ or ‘No’, while questions 2) and 4) provided response options of ‘none’, ‘once’, or ‘twice or more’.

*Tobacco control behaviors of school personnel*

Two questions were asked: In the past year: 1) ‘Have you informed about the harm of tobacco to students?’, and 2) ‘Have you ever stopped students from smoking or persuaded them to quit smoking?’. For each question, respondents could choose ‘Yes’ or ‘No’.

### Statistical analysis

Data analysis was conducted using IBM SPSS 26.0 software. The chi-squared test was used to test the differences between categorical variables. Binary logistic regression was conducted using survey data from 2021 to explore the influencing factors of staff’s tobacco control behaviors. After adjusting for covariates (gender, age group, school type, education level, position, whether had been responsible for work related to health education and whether current smokers), we analyzed the effects of independent variables (the situation of prohibiting students and school personnel from smoking and tobacco control education for school personnel) on dependent variables (tobacco control behaviors of school personnel). The size of the effect was expressed in the form of probability ratios and 95% CI. All statistical analyses were performed using a two-sided hypothesis test, and p<0.05 was considered statistically significant.

## RESULTS

### Characteristics of participants in 2017 and 2021

Table 1 illustrates the characteristics of the sample in both 2017 and 2021. In 2017, 53.47% of the participants were aged ≥40 years, 70.70% were teachers and 88.39% had a Bachelor’s degree or higher. These percentages respectively increased to 57.64%, 73.76% and 93.47% in 2021. Moreover, the proportion of personnel who had been responsible for work related to health education was higher in 2021 (45.60%) than in 2017 (37.70%). The percentages of current smokers decreased from 9.11% in 2017 to 7.16% in 2021. These differences were found to be statistically significant (p<0.05).

**Table 1:** Demographic characteristics of school personnel in 2017 and 2021

*Characteristics*	*Categories*	*2017 (N=2403) n (%)*	*2021 (N=1761) n (%)*	*χ ^2^*	*p*
**Gender**	Male	688 (28.63)	465 (26.41)	2.51	0.113
	Female	1715 (71.37)	1296 (73.59)		
**Age** (years)	<40	1118 (46.53)	746 (42.36)	7.12	0.008
	≥40	1285 (53.47)	1015 (57.64)		
**School type**	Traditional school	1609 (66.96)	1208 (68.60)	1.25	0.264
	Vocational school	794 (33.04)	553 (31.40)		
**Education level**	College or lower	279 (11.61)	115 (6.53)	30.62	<0.001
	Bachelor’s or higher	2124 (88.39)	1646 (93.47)		
**Position**	Teacher	1699 (70.70)	1299 (73.76)	4.73	0.030
	Non-teaching staff	704 (29.30)	462 (26.24)		
**Had been responsible for work related to health education**	Yes	906 (37.70)	803 (45.60)	26.40	<0.001
	No	1497 (62.30)	958 (54.40)		
**Current smokers**	Yes	219 (9.11)	126 (7.16)	7.46	0.024
	No	2184 (90.89)	1635 (92.84)		

### Tobacco control environment in 2017 and 2021

*Smoking and exposure to SHS of school personnel in schools*

As shown in Table 2, the percentages of respondents who ‘smoked on campus in the past year’ and ‘had been exposed to SHS on campus in the past 7 days’ respectively decreased from 4.29% and 23.05% in 2017 to 1.99% and 14.14% in 2021, with decreases of 2.30% and 8.91% (p<0.05).

**Table 2:** Tobacco control environment in 2017 and 2021 in Shanghai

*Characteristics*	*Categories*	*2017 (N=2403) n (%)*	*2021 (N=1761) n (%)*	*χ ^2^*	*p*
**Smoking and exposure to SHS of school personnel in schools**					
Smoked on campus in the past year	Yes	103 (4.29)	35 (1.99)	16.76	<0.001
	No	2300 (95.71)	1726 (98.01)		
Had been exposed to SHS on campus in the past 7 days	Yes	554 (23.05)	249 (14.14)	51.89	<0.001
	No	1849 (76.95)	1512 (85.86)		
**The situation of prohibiting students and school personnel from smoking**					
Agreed the school should strictly prohibit students from smoking	Yes	1904 (79.23)	1482 (84.16)	16.21	<0.001
	No	499 (20.77)	279 (15.84)		
Agreed the school should strictly prohibit personnel from smoking	Yes	1393 (57.97)	1085 (61.61)	5.60	0.018
	No	1010 (42.03)	676 (38.39)		
**Tobacco control education for school personnel**					
Received tobacco control materials from the school	Yes		914 (51.90)		
	No		847 (48.10)		
**Participated in tobacco control trainings**	None	2017 (83.94)	1331 (75.58)	45.30	<0.001
	Once	212 (8.82)	244 (13.86)		
	Twice or more	174 (7.24)	186 (10.56)		
The school had organized tobacco control education activities	Yes	1940 (80.73)	1295 (73.54)	30.35	<0.001
	No	463 (19.27)	466 (26.46)		
Participated in tobacco control education activities	None	1500 (62.42)	1108 (62.92)	1.46	0.481
	Once	567 (23.60)	391 (22.20)		
	Twice or more	336 (13.98)	262 (14.88)		
**Tobacco control behaviors of school personnel**					
Informed about the harm of tobacco to students	Yes		1070 (60.76)		
	No		691 (39.24)		
Stopped students from smoking or persuaded them to quit smoking	Yes	1392 (57.93)	445 (25.27)	439.64	<0.001
	No	1011 (42.07)	1316 (74.73)		

SHS: exposure to secondhand smoke.

*The situation of prohibiting students and school personnel from smoking*

Compared with 79.23% and 57.97% in 2017, the rates of personnel who agreed that ‘the school should strictly prohibit students from smoking’ and ‘the school should strictly prohibit personnel from smoking’ in 2021, increased to 84.16% and 61.61% (p<0.05). In addition, the percentage of staff who agreed that ‘the school should strictly prohibit students from smoking’ was higher than those who agreed that ‘the school should strictly prohibit personnel from smoking’.

*Tobacco control education for school personnel*

In 2021, 51.90% of school personnel reported having ‘received tobacco control materials from the school’. The percentage of personnel who had ‘participated in tobacco control trainings’ in 2017 was 16.06%, rising to 24.42% in 2021. In two surveys, nearly 40% of staff ‘had participated in tobacco control education activities’, but the proportion of staff who knew that ‘the school had organized tobacco control education activities’ in 2021 (73.54%) was lower than that in 2017 (80.73%) (p<0.001).

*Tobacco control behaviors of school personnel*

In 2021, 60.76% of the personnel ‘informed about the harm of tobacco to students’. Those who ‘stopped students from smoking or persuaded them to quit smoking’ significantly decreased from 57.93% in 2017 to 25.27% in 2021 (p<0.001).

### The influencing factors of school personnel’s tobacco control behaviors in 2021

As shown in Table 3, school personnel who worked in vocational schools or who had been responsible for work related to health education were more likely to implement tobacco control behaviors on students than those who worked in traditional schools or who had no experience in health teaching. Moreover, males (AOR=2.18; 95% CI: 1.64–2.90) were more inclined to stop students from smoking or persuade them to quit smoking. Additionally, teachers (AOR=1.39; 95% CI: 1.09–1.78) were more likely to inform about the harm of tobacco to students.

**Table 3:** Impact of demographic characteristics on school personnel’s tobacco control behaviors in 2021 (N=1761)

*Characteristics*	*Categories*	*Informed about the harm of tobacco to students*			*Stopped students from smoking or persuaded them to quit smoking*		
		*%*	*AOR (95% CI)*	*p*	*%*	*AOR (95% CI)*	*p*
**Gender**	Female ®	59.88	1		20.99	1	
	Male	63.23	1.20 (0.93–1.56)	0.163	37.20	2.18 (1.64–2.90)	<0.001
**Age** (years)	<40 ®	60.00	1		26.80	1	
	≥40	61.80	1.05 (0.86–1.30)	0.628	23.19	1.28 (0.99–1.65)	0.059
**School type**	Traditional school ®	56.95	1		13.66	1	
	Vocational school	69.08	2.09 (1.67–2.63)	<0.001	50.63	7.69 (5.94–9.94)	<0.001
**Education level**	College or lower ®	46.09	1		33.04	1	
	Bachelor’s or higher	61.79	0.75 (0.48–1.16)	0.193	24.73	1.22 (0.72–2.05)	0.465
**Position**	Non-teaching staff ®	54.11	1		25.54	1	
	Teachers	63.13	1.39 (1.09–1.78)	0.007	25.17	1.29 (0.96–1.73)	0.096
**Had been responsible for work related to health education**	No ®	51.98	1		24.22	1	
	Yes	71.23	2.46 (2.00–3.03)	<0.001	26.53	1.65 (1.28–2.12)	<0.001
**Smoked in the past 30 days**	No ®	60.92	1		24.46	1	
	Yes	58.73	1.06 (0.68–1.65)	0.797	35.71	0.87 (0.54–1.41)	0.578

AOR: adjusted odds ratio. AORs (95% CI) were calculated using a binary logistic regression model. Gender, age group, school type, education level, position, whether had been responsible for work related to health education and whether to be current smokers were included in the model through input methods. ® Reference categories.

As presented in Table 4, those who agreed that the school should strictly prohibit students from smoking (AOR=1.64; 95% CI: 1.25–2.15), received tobacco control materials from the school (AOR=2.96; 95% CI: 2.41–3.64), knew that the school had organized tobacco control education activities (AOR=4.29; 95% CI: 3.39–5.42) and participated in trainings and education activities of tobacco control, were more likely to inform about the harm of tobacco to students. And the more frequently they participated in tobacco control trainings or education activities, the more likely they were to actively inform about the harm of tobacco to students.

**Table 4:** The impact of tobacco control environment on tobacco control behaviors of school personnel in 2021 (N=1761)

*Variables*	*Categories*	*Informed about the harm of tobacco to students*			*Stopped students from smoking or persuaded them to quit smoking*		
		*%*	*AOR (95% CI)*	*p*	*%*	*AOR (95% CI)*	*p*
**Agreed the school should strictly prohibit students from smoking**	No ®	53.76	1		49.10	1	
	Yes	62.08	1.64 (1.25–2.15)	<0.001	20.78	0.30 (0.22–0.41)	<0.001
**Agreed the school should strictly prohibit personnel from smoking**	No ®	59.02	1		34.91	1	
	Yes	61.84	1.13 (0.92–1.38)	0.257	19.26	0.46 (0.36–0.59)	<0.001
**Received tobacco control materials from the school**	No ®	46.87	1		22.08	1	
	Yes	73.63	2.96 (2.41–3.64)	<0.001	28.23	1.08 (0.84–1.37)	0.557
Participated in tobacco control trainings	0 ®	53.34	1		22.31	1	
	Once	80.33	3.12 (2.21–4.39)	<0.001	29.10	1.26 (0.90–1.77)	0.185
	Twice or more	88.17	5.37 (3.37–8.56)	<0.001	41.40	1.87 (1.30–2.69)	0.001
**The school had organized tobacco control education activities**	No ®	33.48	1		19.74	1	
	Yes	70.58	4.29 (3.39–5.42)	<0.001	27.26	1.20 (0.90–1.60)	0.213
**Participated in tobacco control education activities**	0 ®	48.10	1		19.58	1	
	Once	79.28	3.83 (2.90–5.07)	<0.001	32.99	2.06 (1.54–2.75)	<0.001
	Twice or more	86.64	5.90 (4.02–8.67)	<0.001	37.79	1.98 (1.42–2.75)	<0.001

AOR: adjusted odds ratio. AORs (95% CI) were calculated using a binary logistic regression model, adjusted for gender, age group, school type, education level, position, whether had been responsible for work related to health education and whether to be current smokers. ® Reference categories.

Similarly, school staff who participated in tobacco control education activities and twice or more times in tobacco control trainings (AOR=1.87; 95% CI: 1.30–2.69) were more inclined to stop students from smoking or to persuade them to quit smoking. Furthermore, staff who thought that the school should strictly prohibit students (AOR=0.30; 95% CI: 0.22–0.41) and personnel (AOR=0.46; 95% CI: 0.36–0.59) from smoking, were prone to stop students from smoking or persuade them to quit smoking.

## DISCUSSION

This study revealed that the enforcement of smoking bans in Shanghai ‘s secondary schools, aimed at prohibiting both students and personnel from smoking, had significantly strengthened in 2021 compared to 2017. Nevertheless, despite this progress, certain challenges persisted within these schools, notably exposure to secondhand smoke (SHS), inadequate anti-smoking measures, and a scarcity of tobacco control education for school personnel. School personnel could play an active role in tobacco control work on campus, with teachers and staff who had been responsible for work related to health education or received education on tobacco control being more likely to engage in tobacco control behaviors with students.

Compared with 2017, there was an increased approval among staff in Shanghai’s secondary schools for strict smoking prohibition policies that apply to both students and personnel in 2021. Meanwhile, smoking rates and SHS exposure rates in schools decreased, which aligned with a survey conducted by China CDC that reported reduced percentages of secondary school students observing teachers smoking almost every day on campus compared to 2019^28^. In addition, some schools had intensified their tobacco control education for staff, with a higher percentage of staff participating in tobacco control trainings in 2021 compared to 2017. These changes underscore the effectiveness of the series of school tobacco control policies implemented in China from 2017 to 2021.

However, the study also identified that some staff members contributed to exposure to SHS in schools, suggesting that Shanghai’s tobacco control policies on campus were not fully implemented across all institutions. Consequently, it is imperative to strengthen tobacco control policies and rigorously address SHS exposure on campus in the future. In schools where tobacco control policies were not strictly enforced, smoking rates among staff and students tended to be higher^12^, indicating that these schools’ personnel did not have opportunities to intervene and discourage smoking among students. These staff members were less likely to inform students of the harmful effects of tobacco, as evidenced by previous research^22,25,26^. Furthermore, there were several issues pertaining to tobacco control policies in Shanghai’s secondary schools, including a lack of specific systems, vacancies of functional departments and inadequate implementation of tobacco control measures. To ensure the effective enforcement of tobacco control policies on campus, more diverse measures, such as establishing more functional departments for tobacco control or hiring external professional supervisors, should be considered and tailored to the specific circumstances^17^.

In this study, staff perceived that the tobacco control policies for themselves were less stringent than those for students, a finding that concurred with the Global School Personnel Survey (GSPS)^29^. Both student and personnel tobacco control policies were found to be associated with lower rates of teenagers smoking attempts and smoking within school^12,30^. Staff members’ smoking behavior on-campus not only had a direct influence on adolescent smoking^14^, but also gave rise to disparities in policy implementation, making it challenging for students to internalize anti-smoking personal beliefs and, consequently, indirectly leading to adolescents smoking^18^. Therefore, tobacco control efforts should encompass all individuals in schools, including school personnel, with stricter measures to prohibit smoking by staff members on campus; for instance, making non-smoking during school hours a condition of employment for staff ^15^, or including staff smoking behavior on campus a factor for deduction in performance assessment.

The tobacco control behaviors of staff were closely related to the level of tobacco control education provided by schools. Staff members who had received tobacco control materials and participated in tobacco control trainings exhibited stronger awareness of anti-smoking and were more likely to adopt tobacco control behaviors with students^25,26,31^. However, in 2021, only 51.90% of Shanghai secondary school staff received tobacco control materials, a value lower than those reported in the EU (57.4%) and certain regions of Pakistan (60.9–86.4%)^29^. Furthermore, only 24.42% of staff participated in tobacco control trainings in 2021, far below their tobacco control training requirements (78.02%)^32^. This finding aligned with previous research, which indicated that the proportions of school personnel trained in tobacco control (7–27%) across all regions were consistently lower than their training requirements (38–85%)^29^.

In addition, engaging in various forms of tobacco control education activities, such as class meetings and knowledge competitions, may not only improve teenagers’ awareness of tobacco control knowledge^33,34^, but also promote the development of tobacco control behaviors among school personnel. However, only 37.08% of school personnel participated in tobacco control education activities in 2021, and the percentage of those who knew that the school had organized such activities showed a downward trend. A study had pointed out that the main reasons for staff to implement the school tobacco control policies included perceiving it as part of their responsibilities, believing in the positive effects of tobacco control, and having enough confidences in handling students’ reactions^35^. Therefore, to enhance staff’s responsibility for tobacco control, schools should organize diverse tobacco control education activities and encourage active participation among staff members^12^.

Moreover, the smoking rate of vocational school students was found to be higher than that of traditional school students^12,36^. Vocational schools’ staff were more inclined to participate in tobacco control trainings or educational activities and adopt tobacco control behaviors with students. School personnel who had been responsible for work related to health education were likely to engage in tobacco control behaviors, consistent with previous findings that such personnel were more prone to advising students to quit smoking^22^. In 2021, 45.60% of staff participated in health teaching related work. Encouraging more staff to be involved in health teaching related work could enhance the health literacy of school personnel and foster a favorable environment for tobacco control on campus^22,25^. Besides, compared with non-teaching staff, teachers were more inclined to inform about the harm of tobacco to students, possibly due to greater contact between teachers and students, which facilitated the easier detection of students’ smoking behavior. Given the potential role of non-teaching staff in tobacco control, it is essential to strengthen their tobacco control training and mobilize them^25^.

### Limitations

Some limitations should be acknowledged in this study. First of all, the respondents were limited to school personnel in secondary schools in Shanghai. The differences in economic conditions and lifestyle made it difficult to directly generalize the research results to other countries and regions. Secondly, the cross-sectional study could only preliminarily explore the correlation between the school tobacco control environment and staff’s tobacco control behaviors, lacking the ability to determine causal relationships between them. Thirdly, due to the anonymously self-report nature of the questionnaires, reports on school tobacco control environment might be over- or under-estimated. Fourthly, the COVID-19 pandemic may have influenced the smoking behavior of school personnel, but its impact was unclear and therefore not discussed in this survey. Therefore, future research should include intervention studies involving diverse populations in multiple regions with varying economic conditions and lifestyles to explore more accurate causal relationships.

## CONCLUSIONS

The study revealed that the tobacco control environment in Shanghai’s secondary schools had significantly improved in 2021 compared with 2017. The reduction in smoking and SHS exposure among staff underscores the effectiveness of the tobacco control policies implemented in China from 2017 to 2021. However, challenges remain such as inadequate smoking control education for school personnel. Comprehensive tobacco control efforts should encompass everyone in the school, not just students. Regular distribution of tobacco control materials and active organization of tobacco control trainings or education activities can strengthen school personnel’s awareness of tobacco control and encourage them to adopt proactive tobacco control behaviors, thereby contributing to the construction and development of smoke-free schools.

## Data Availability

The data supporting this research are available from the authors on reasonable request.

## References

[1] GBD 2019 Tobacco Collaborators. Spatial, temporal, and demographic patterns in prevalence of smoking tobacco use and attributable disease burden in 204 countries and territories, 1990-2019: a systematic analysis from the Global Burden of Disease Study 2019. Lancet. 2021;397(10292):2337-2360. doi:10.1016/S0140-6736(21)01169-7 34051883 PMC8223261

[2] Park-Lee E, Ren C, Cooper M, Cornelius M, Jamal A, Cullen KA. Tobacco product use among middle and high school students - United States, 2022. MMWR Morb Mortal Wkly Rep. 2022;71(45):1429-1435. doi:10.15585/mmwr.mm7145a1 36355596 PMC9707354

[3] Jiang LL, Liu HY, Ma ZJ, Wang Y. A comparative study on tobacco epidemic among middle school students in Heilongjiang Province in 2014 and 2019. Article in Chinese. Chinese Journal of Health Education. 2022;38(7):589-592.

[4] Miao WF. The health risks of smoking for adolescents. Article in Chinese. Journal of Clinical Medical Literature. 2017;4(47):9306. doi:10.16281/j.cnki.jocml.2017.47.148

[5] Chang G, Sherritt L, Knight JR. Adolescent cigarette smoking and mental health symptoms. J Adolesc Health. 2005;36(6):517-522. doi:10.1016/j.jadohealth.2004.05.008 15901517

[6] U.S. National Center for Chronic Disease Prevention and Health Promotion. Office on Smoking and Health. Preventing Tobacco Use Among Youth and Young Adults: A Report of the Surgeon General. U.S. Centers for Disease Control and Prevention; 2012. Accessed July 29, 2024. https://www.ncbi.nlm.nih.gov/books/NBK99237/22876391

[7] World Health Organization. Hooking the next generation: how the tobacco industry captures young customers. World Health Organization; 2024. Accessed July 29, 2024. https://iris.who.int/bitstream/handle/10665/376853/9789240094642-eng.pdf?sequence=1

[8] Guan QY, Zhu WH, Mai JR. Research progress on current situation of e-cigarette use and its influencing factors among teenager. Article in Chinese. Chinese Journal of Health Education. 2022;38(1):81-84.

[9] Fadus MC, Smith TT, Squeglia LM. The rise of e-cigarettes, pod mod devices, and JUUL among youth: factors influencing use, health implications, and downstream effects. Drug Alcohol Depend. 2019;201:85-93. doi:10.1016/j.drugalcdep.2019.04.011 31200279 PMC7183384

[10] Wang TW, Gentzke AS, Creamer MR, et al. Tobacco product use and associated factors among middle and high school students - United States, 2019. MMWR Surveill Summ. 2019;68(12):1-22. doi:10.15585/mmwr.ss6812a1 PMC6903396 31805035

[11] MacArthur G, Caldwell DM, Redmore J, et al. Individual-, family-, and school-level interventions targeting multiple risk behaviours in young people. Cochrane Database Syst Rev. 2018;10(10):CD009927. doi:10.1002/14651858.CD009927.pub2 30288738 PMC6517301

[12] Mlinarić M, Günther S, Moor I, Winter K, Hoffmann L, Richter M. Der Zusammenhang zwischen schulischer Tabakkontrolle und der wahrgenommenen Raucherprävalenz Jugendlicher. The association between school tobacco policies and the perceived smoking prevalence of adolescents. Article in German. Bundesgesundheitsblatt Gesundheitsforschung Gesundheitsschutz. 2021;64(1):91-101. doi:10.1007/s00103-020-03261-1 33284361 PMC7772164

[13] Chen SX, Hu Y, Huang X, Liu JC. Study on the prevalence of tobacco exposure among adolescents in Haikou City,2019. Article in Chinese. Chinese Journal of Health Education. 2022;38(10):927-931.

[14] Backhaus I, D’Egidio V, Grassucci D, Gelardini M, Ardizzone C, La Torre G. Compliance with the school smoking ban: a cross-sectional study from Italy. Clin Ter. 2021;172(2):138-144. doi:10.7417/CT.2021.2301 33763668

[15] Schreuders M, van den Putte B, Kunst AE. Smoke-free school policies in Europe: challenges for the future. Prev Med. 2020;138:106130. doi:10.1016/j.ypmed.2020.106130 32439487

[16] Nguyen VH, Do DA, Do TTH, et al. Smoke-free environment policy in Vietnam: what did people see and how did they react when they visited various public places? J Prev Med Hyg. 2019;60(1):E36-E42. doi:10.15167/2421-4248/jpmh2019.60.1.942 31041409 PMC6477555

[17] Galanti MR, Coppo A, Jonsson E, Bremberg S, Faggiano F. Anti-tobacco policy in schools: upcoming preventive strategy or prevention myth? A review of 31 studies. Tob Control. 2014;23(4):295-301. doi:10.1136/tobaccocontrol-2012-050846 23716172

[18] Kim SY, Jang M, Yoo S, JeKarl J, Chung JY, Cho SI. School-based tobacco control and smoking in adolescents: evidence from multilevel analyses. Int J Environ Res Public Health. 2020;17(10):3422. doi:10.3390/ijerph17103422 32423028 PMC7277168

[19] Azagba S, Kennedy RD, Baskerville NB. Smoke-free school policy and exposure to secondhand smoke: a quasi-experimental analysis. Nicotine Tob Res. 2016;18(2):170-176. doi:10.1093/ntr/ntv077 25847291 PMC4710206

[20] Agaku IT, Singh T, Rolle I, Olalekan AY, King BA. Prevalence and determinants of secondhand smoke exposure among middle and high school students. Pediatrics. 2016;137(2):e20151985. doi:10.1542/peds.2015-1985 26755696

[21] Jang BN, Jeong W, Kang SH, Jang SI. Association between the location of secondhand smoke exposure and depressive symptoms among South Korean adolescents. Int J Environ Res Public Health. 2020;17(14):5116. doi:10.3390/ijerph17145116 32679863 PMC7400535

[22] Chen PL, Huang WG, Chao KY. Factors associated with Taiwanese junior high school personnel advising students to quit smoking. J Sch Health. 2011;81(2):91-99. doi:10.1111/j.1746-1561.2010.00565.x 21223276

[23] Zhang W, Long XH, Xiao LZ, Liang HH, Luo KM. Intervention effects of tobacco control health education on smoking perceptions and tobacco use attitudes of secondary school boys. Article in Chinese. Health Vocational Education. 2019;37(19):125-127.

[24] Zhu JF, He YP, Li N, Cai Y, Tao JJ, Ma J. Prevalence of smoking and tobacco control environment among middle school personnel in Shanghai. Article in Chinese. Journal of Shanghai Jiaotong University (Medical Science). 2012;32(7):857-860. Accessed July 29, 2024. https://xuebao.shsmu.edu.cn/EN/10.3969/j.issn.1674-8115.2012.07.008

[25] Qin R, Guo X, Xu W, Ma Y. Analysis of smoking surveillance results among Beijing primary and secondary school staff in 2019. Chinese Journal of School Health. 2021;42(5):683-688. doi:10.16835/j.cnki.1000-9817.2021.05.011

[26] Tan YL, Chen ZY, He YP, Xu G, Yu ZP, Zhu JF. Awareness of tobacco control policies and anti-tobacco attitudes and behaviors among school personnel. Tob Induc Dis. 2022;20(June):54. doi:10.18332/tid/149926 35799622 PMC9179323

[27] Dunlop S, Kite J, Grunseit AC, et al. Out of sight and out of mind? Evaluating the impact of point-of-sale tobacco display bans on smoking-related beliefs and behaviors in a sample of Australian adolescents and young adults. Nicotine Tob Res. 2015;17(7):761-768. doi:10.1093/ntr/ntu180 25283169

[28] People's Daily Health Client. Tobacco Epidemic Surveillance Results Released for Chinese High School and University Students in 2021. In Chinese. Baidu. May 31, 2022. Accessed May 29, 2022. https://baijiahao.baidu.com/s?id=1734321347492573592&wfr=spider&for=pc

[29] GTSS Collaborative Group. The Global School Personnel Survey: a cross-country overview. Tob Control. 2006;15 Suppl 2(Suppl 2):ii20-ii30. doi:10.1136/tc.2006.015693 16731521 PMC2563538

[30] Jayawardhana J, Bolton HE, Gaughan M. The association between school tobacco control policies and youth smoking behavior. Int J Behav Med. 2019;26(6):658-664. doi:10.1007/s12529-019-09825-z 31741294

[31] Mermer G, Dağhan Ş, Bilge A, Dönmez RÖ, Özsoy S, Günay T. Prevalence of tobacco use among school teachers and effect of training on tobacco use in Western Turkey. Cent Eur J Public Health. 2016;24(2):137-143. doi:10.21101/cejph.a4217 27434246

[32] Tan YL, He YP, Chen ZY, Yang XC, Zhu JF. Participation in school tobacco control work and demand for tobacco control training among middle school faculties in Shanghai. Article in Chinese. Chinese Journal of Health Education. 2021;37(04):308-311, 337.

[33] Qin J, Tan ZL, Zhao JN, Jia G. Evaluation on effect of tobacco-control theme class meeting on improving tobacco control knowledge and behavior of primary school students. Preventive Medicine Tribune. 2020;(8):595-597, 601.

[34] Andersen S, Rod MH, Ersbøll AK, et al. Effects of a settings-based intervention to promote student wellbeing and reduce smoking in vocational schools: a non-randomized controlled study. Soc Sci Med. 2016;161:195-203. doi:10.1016/j.socscimed.2016.06.012 27319278

[35] Linnansaari A, Schreuders M, Kunst AE, Rimpelä A, Lindfors P. Understanding school staff members’ enforcement of school tobacco policies to achieve tobacco-free school: a realist review. Syst Rev. 2019;8(1):177. doi:10.1186/s13643-019-1086-5 31324212 PMC6642528

[36] Zhu J, Ma J, Li N, et al. Z-analysis on the factors affecting smoking behavior and future smoking intention of adolescents in Shanghai. Chinese Journal of School Health. 2017;38(10):1478-1481. doi:10.16835/j.cnki.1000-9817.2017.10.012

